# Targeted gene deletions in the dimorphic fungal pathogen *Histoplasma* using an optimized episomal CRISPR/Cas9 system

**DOI:** 10.1128/msphere.00178-23

**Published:** 2023-06-30

**Authors:** Chad A. Rappleye

**Affiliations:** 1 Department of Microbiology, Ohio State University, Columbus, Ohio, USA; University of Georgia, Athens, Georgia, USA

**Keywords:** *Histoplasma*, CRISPR, genome editing

## Abstract

The rapid development of CRISPR/CRISPR-associated (Cas) systems has revolutionized the ability to produce genetic mutations in a desired locus, particularly in organisms with low rates of homologous recombination. *Histoplasma* is an important respiratory and systemic fungal pathogen that has few reverse genetic options. We describe an optimized CRISPR/Cas system for the efficient generation of mutations in desired genes. The limited requirements for CRISPR/Cas, namely a gene-targeting guide RNA (gRNA) and expression of a Cas endonuclease, enabled both the gRNA and the *Streptococcus pyogenes* Cas9 gene to be expressed from a single episomal vector. The gRNAs are expressed from a strong Pol(II) promoter, a critical parameter for increasing the recovery of mutated genes, and processed into the mature gRNA by ribozymes in the mRNA. Expression of dual-tandem gRNAs facilitates the generation of gene deletions at a good frequency which can be detected by PCR-based screening of pooled isolates resulting in the isolation of marker-less deletion mutants. The CRISPR/Cas system is encoded on an episomal telomeric vector facilitating curing strains of the CRISPR/Cas vector upon generation of the mutant. We demonstrate the successful application of this CRISPR/Cas system in diverse *Histoplasma* species and applicable for multiple genes. The optimized system shows promise for accelerating reverse genetic studies in *Histoplasma* spp.

**IMPORTANCE:**

The ability to eliminate gene product functions is central to understanding molecular mechanisms. In the fungal pathogen *Histoplasma*, methods to inactivate or deplete gene products are inefficient, which hampers progress in defining *Histoplasma*’s virulence mechanisms. We describe an efficient CRISPR/Cas-based system for generating gene deletions in *Histoplasma* and show its validation on multiple genes with selectable and non-selectable phenotypes.

## INTRODUCTION

One of the critical methods to demonstrate the function of a gene product is through loss-of-function genetic studies. The ability to create or generate mutants varies widely among microorganisms and model systems. High native rates of homologous recombination enable directed mutations to be generated with relative ease, but homologous recombination rates can be extremely low in some organisms, frustrating reverse genetic approaches.

The discovery of the clustered regularly interspaced short palindromic repeat (CRISPR) system of bacterial immunity (1 - 3) has revolutionized genome editing in numerous organisms, particularly those with low homologous recombination rates. CRISPR-associated (Cas) endonucleases are guided to target sequences in DNA by CRISPR RNAs (crRNA) in complex with a transactivating RNA (tracrRNA) (4, 5). The 20-nucleotide protospacer element in the crRNA provides specificity for the subsequent DNA cleavage by the Cas endonuclease. Genome editing via the CRISPR/Cas system relies on DNA repair processes in the host organism, creating small insertion/deletion events (indels) at the targeted cut site to cause mutation of the target locus or through double-strand break-induced homologous recombination with an edited template molecule. The CRISPR/Cas system has been further engineered to enable crRNA-based targeting for activation of gene transcription (CRISPRa) or inhibition of gene expression (CRISPRi) using engineered Cas proteins (6 - 8). The relatively few components required for CRISPR-based genome editing combined with the ability to induce mutations without reliance on homologous recombination make the CRISPR/Cas system attractive for reverse genetics in difficult organisms.

*Histoplasma* species are important fungal pathogens with limited options for creating loss-of-function alleles. *Histoplasma* has recently been designated a World Health Organization (WHO) priority fungal pathogen (9), raising the need for molecular genetic tools to enable studies of *Histoplasma*’s pathogenic mechanisms. Beyond studies of pathogenesis (10 - 12), the thermal dimorphism of *Histoplasma* is a system to investigate the regulation of fungal morphology and cellular differentiation (13 - 15). A procedure to create gene knockouts in *Histoplasma* was developed in 2000 (16), leading to the isolation of the first directed mutation. However, the process is inefficient due to extensive time required, low success rates, and is largely limited to a single species. Consequently, alternative means to eliminate gene functions were developed including RNA-interference (RNAi) (17, 18) and insertion of an engineered T-DNA element of *Agrobacterium* (19 - 21). Although greater success in loss-of-function studies was realized with these methods, gene depletion by RNAi can be unstable and T-DNA insertional mutagenesis cannot be targeted to a locus of interest.

To overcome the existing limitations of current technologies for loss-of-function studies in *Histoplasma* and related dimorphic fungi, there has been considerable interest in and early work to apply CRISPR/Cas genome editing (22). We describe an optimized system for the expression of gene targeting crRNAs by Pol(II)-based transcription of a single chimeric guide RNA (gRNA or sometimes referred to as sgRNA) consisting of the crRNA and tracrRNA in one molecule. The gRNA processing from the transcription product is based on dual ribozyme-based cleavage of the mRNA, a method designed for precise RNA processing and used in a variety of fungi (23 - 26). Both the gRNA and the Cas9 endonuclease are carried on a *Histoplasma* episomal plasmid facilitating removal of the CRISPR/Cas9 system, following realization of a mutational event. We demonstrate the efficiency of the system in generating mutations in multiple genes and show the broad applicability of the system to diverse phylogenetic lineages [species; reference (27)] of *Histoplasma.*

## RESULTS

### Expression of gRNAs and Cas9 causes mutation of targeted genes

**Fig 1:**
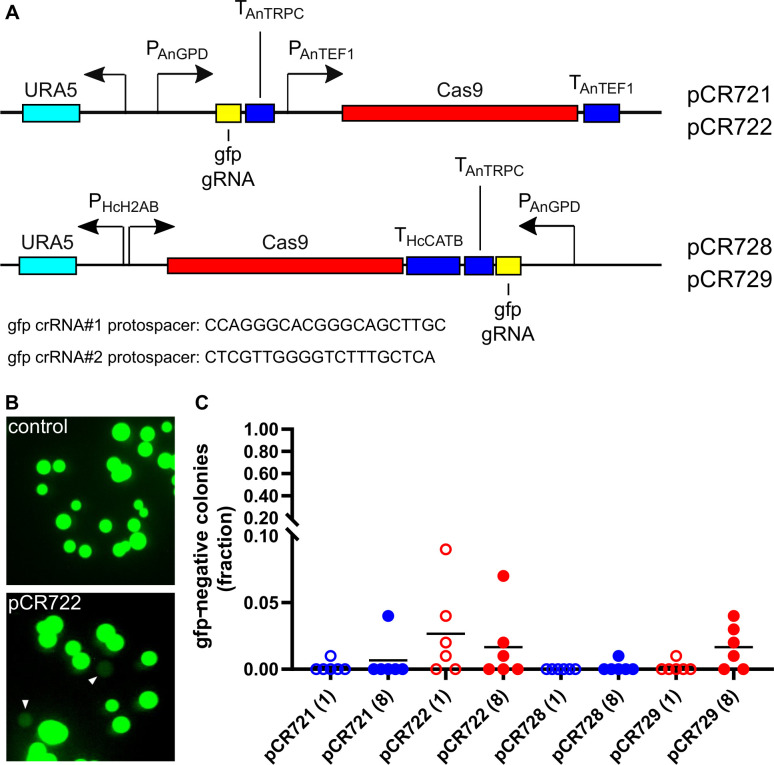
CRISPR-based genome editing induces mutation of a GFP target gene. (A) Schematic of vectors for expression of Cas9 and GFP-targeting gRNAs. Vectors were based on CRISPR systems of Kujoth et al. (pCR721 and pCR722) or Rodriquez et al. (pCR728 and pCR729) on which *S. pyogenes* Cas9 and GFP-targeting gRNAs were placed. Gene expression regulatory elements of each vector are indicated [*Aspergillus* GAPDH promoter (P_AnGPD_), *Aspergillus TRPC* terminator (T_AnTRPC_), *Aspergillus TEF1* promoter (P_AnTEF1_), *Aspergillus TEF1* terminator (T_AnTEF1_), the bidirectional *Histoplasma H2AB* promoter (P_HcH2AB_), and the *Histoplasma CATB* terminator (T_HcCATB_)]. The protospacer sequence in the crRNAs designed to guide Cas9 endonuclease cleavage of GFP is listed and each incorporated into both vectors (crRNA#1: pCR721 and pCR728; crRNA#2: pCR722 and pCR729). (B and C) Quantitation of the loss of GFP fluorescence due to transformation of yeasts with CRISPR/Cas vectors. (B) Image of colonies derived from yeasts transformed with an empty CRISPR/Cas vector (control; top) or a GFP-targeting CRISPR/Cas vector (pCR722, bottom) shows loss of GFP fluorescence (arrowheads) with GFP-targeting gRNAs. (C) Quantification of the frequency of loss of GFP fluorescence in colonies with vectors containing crRNA#1 (blue) and crRNA#2 (red). Clonal lines were established from transformed yeast and plated immediately (one passage, open symbols) or after eight passages in a liquid medium (closed symbols), and the number of GFP-negative colonies was determined. Each data point represents the frequency of GFP-negative colonies from a single line (*n* >25 colonies for each line). Horizontal bars represent the mean fraction of GFP-negative colonies (*n* = 6 independent lines).

To determine if expression of gRNAs could facilitate mutation of genes, we expressed two different gRNAs targeting GFP in GFP-expressing *Histoplasma* yeasts. Targeting GFP provided an efficient and quantitative means of assessing mutational events by the loss of GFP fluorescence in yeast colonies on solid media. Two different CRISPR/Cas9 systems have been used in dimorphic fungi (26, 28), both of which express the gene targeting gRNA and the *Streptococcus pyogenes* Cas9 transgene from a single vector construct. In the system of Kujoth et al. (26), all gene expression regulatory elements are derived from *Aspergillus nidulans*, which have been successfully used in *Blastomyces dermatitidis,* an Onygenales species closely related to *Histoplasma*. In both systems, Cas9 expression is driven by a constitutive promoter (the *Aspergillus TEF1* promoter or the *Histoplasma H2B* promoter) and the gRNA expressed from the *Aspergillus* GAPDH promoter. To facilitate transformation into *Histoplasma* yeasts, both CRISPR/Cas systems were moved onto telomeric vectors (data not shown; Bastian Joehnk personal communication) (29). To quantitatively compare the efficiency of the two systems in targeting a gene in *Histoplasma*, GFP was selected as the target since loss of GFP could be easily tracked by loss of fluorescence. Two 20-nucleotide GFP-targeting protospacers were incorporated into the gRNA modules of each system, which were placed on a *URA5*-containing plasmid to enable transformation and selection in *Histoplasma* (Fig. 1A).

Transformation of each construct into GFP-fluorescent *Histoplasma* yeasts resulted in little to no loss of GFP fluorescence in transformant colonies (Fig. 1B). Since CRISPR/Cas-dependent mutation is dependent on the inaccurate repair of the cut DNA, transformed yeasts were also passaged eight times in liquid culture to allow additional cut/repair events before plating on solid media. For both gRNAs in both CRISPR/Cas9 systems, passaging the yeasts in this way increased the frequency of GFP-negative colonies, although the mean frequency still remained below 5% (Fig. 1C). Transformation of yeasts with a non-CRISPR/Cas9 plasmid did not produce non-fluorescent colonies (Fig. 1B; “control”) indicating that the loss of fluorescence was due to the CRISPR/Cas9 system. Sequence analysis of the GFP gene in pools of the transformants showed a low frequency of indel mutations (data not shown), confirming the gene editing. While both systems produced non-fluorescent mutants, the *Histoplasma* system based on the vector of Kujoth et al. proved slightly superior in the generation of mutants. In both systems, the GFP-targeting crRNA#2 was more effective (Fig. 1C).

### Increased expression of the gRNA increases the frequency of gene editing

**Fig 2:**
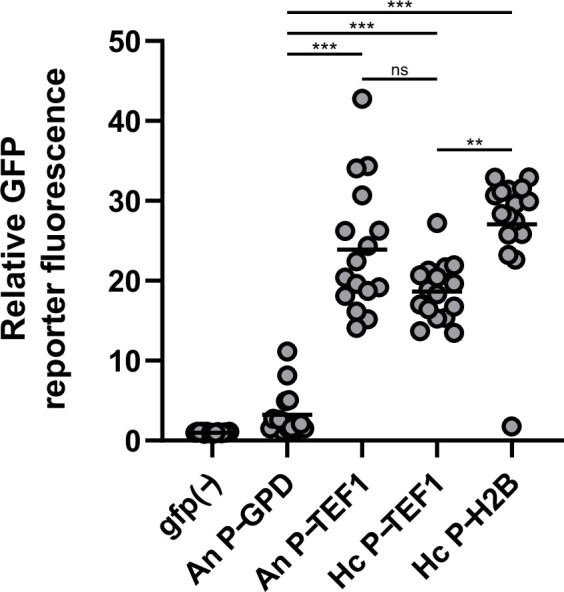
Relative transcription levels of *Aspergillus* and *Histoplasma* promoters in *Histoplasma* yeasts. *Histoplasma* yeasts were transformed with promoter-GFP transcriptional fusions with *Aspergillus* promoters derived from the CRISPR vector of Kujoth et al., the *Histoplasma* constitutive *TEF1* promoter (Hc P_TEF1_), or the *Histoplasma H2B* promoter used in the system of Rodriguez et al. *Histoplasma* yeasts were transformed with each vector, and the fluorescence of individual transformant colonies was measured. Data points represent the GFP fluorescence of individual transformant colonies relative to *Histoplasma* yeast lacking GFP [GFP(−)] with horizontal lines indicating the mean GFP fluorescence (*n* = 16). Significant differences in promoter strength based on ANOVA with Tukey’s multiple correction tests are indicated by asterisks (ns, non-significant; **P < 0.01; and ***P < 0.001).

The observed frequency of mutation with the above systems is too low for effective gene editing, particularly for genes that do not produce visible phenotypes with loss of function mutations. While both CRISPR/Cas9 systems use the same general design, including expression of the gRNA and Cas9, the major differences in the two systems are the transcriptional regulatory elements (e.g., promoters and transcriptional terminators). The gRNAs were commonly expressed from the *Aspergillus* GAPDH promoter. To test the efficiency of the different promoters employed for Cas9 and gRNA expression, each promoter was cloned into a GFP transcriptional reporter vector and transformed into non-fluorescent *Histoplasma* yeast. As a control, a similar construct using the established *Histoplasma TEF1* promoter was also tested. All promoters were sufficient to drive expression in *Histoplasma* yeast, but the *Aspergillus* GAPDH promoter was much weaker than the *Aspergillus* or *Histoplasma TEF1* promoters or the *Histoplasma H2B* promoter (Fig. 2).

**Fig 3:**
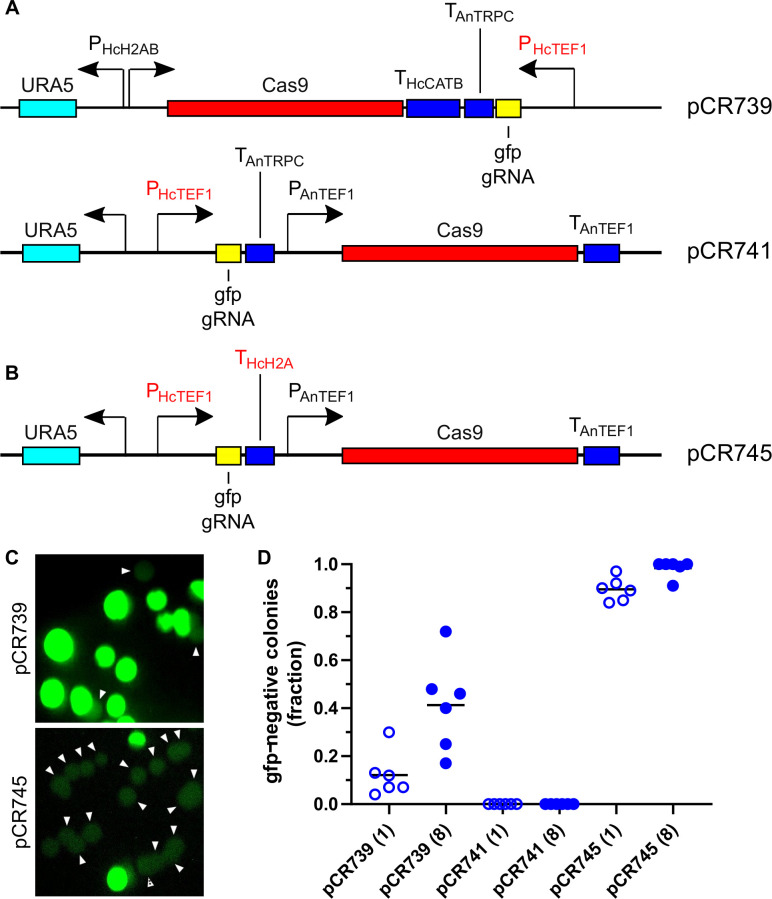
Optimization of transcriptional control elements on CRISPR/Cas vectors improves the efficiency of gene mutation. (A and B) Schematic representation of CRISPR/Cas vectors targeting GFP with gRNAs driven by the *Histoplasma TEF1* promoter (P_HcTEF1_; red). In both vectors, the gRNA harbors GFP-targeting crRNA#2. (A) The *Aspergillus* GAPDH promoter controlling gRNA expression was replaced by the *Histoplasma TEF1* promoter. (B) The *Aspergillus TRPC* terminator was replaced with the putative *Histoplasma H2A* terminator (T_HcH2A_; red). (C and D) Quantitation of the loss of GFP fluorescence due to transformation of yeasts with CRISPR/Cas vectors. (C) Image of colonies derived from yeasts transformed with GFP-targeting CRISPR/Cas vectors (pCR739 and pCR745, top and bottom, respectively) shows loss of GFP fluorescence (arrowheads) with GFP-targeting gRNAs. (D) Quantification of the frequency of loss of GFP fluorescence in colonies with vectors in which expression of the GFP-targeting gRNA is driven by the *Histoplasma TEF1* promoter. Clonal lines were established from transformed yeast and plated immediately (one passage, open symbols) or after eight passages in a liquid medium (closed symbols), and the number of GFP-negative colonies was determined. Each data point represents the fraction of GFP-negative colonies from a single line (*n* >25 colonies for each line). Horizontal bars represent the mean fraction of GFP-negative colonies (*n* = 6 lines).

The weak *Aspergillus* GAPDH promoter was replaced by the *Histoplasma TEF1* promoter in each CRISPR/Cas system to determine if a higher expression of the gRNAs would increase the gene editing frequency (Fig. 3A). Expression of GFP-targeting gRNAs from this stronger promoter dramatically increased generation of non-fluorescent transformants in the CRISPR/Cas system based on Rodriguez et al. (pCR739; Fig. 3B), and additional passaging of cells further increased mutation frequency (Fig. 3C). Surprisingly, replacement of the *Aspergillus* GAPDH promoter with the *Histoplasma TEF1* promoter in the system of Kujoth et al. (pCR741; Fig. 3C) failed to produce any non-fluorescent *Histoplasma* yeasts in contrast to the system with the *Aspergillus* GAPDH promoter used earlier (pCR721/722; Fig. 1B). In vector pCR741, transcription of the gRNA and the Cas9 gene is arranged in the same direction with an intervening *Aspergillus TRPC* terminator region. We reasoned that due to the common direction of transcription, readthrough transcription driven by the strong *Histoplasma TEF1* promoter could impair transcription initiation of the downstream Cas9 gene, if the intervening exogenous terminator sequence was weak or non-functional. To test this, we replaced the *Aspergillus TRPC* terminator with the intergenic region between the highly transcribed *Histoplasma H2A* gene and the next gene encoded in the genome, reasoning that this region would include the necessary transcription termination signals to end transcripts originating from the strong *H2A* promoter. Inclusion of this putative *H2A* transcription terminator region (Fig. 3B) dramatically improved the frequency of obtaining non-fluorescent *Histoplasma* yeasts, with an average of 90% effectiveness, even without passaging the transformants (pCR745; Fig. 3B and C).

### Expression of dual sgRNAs generates deletions in target genes

**Fig 4:**
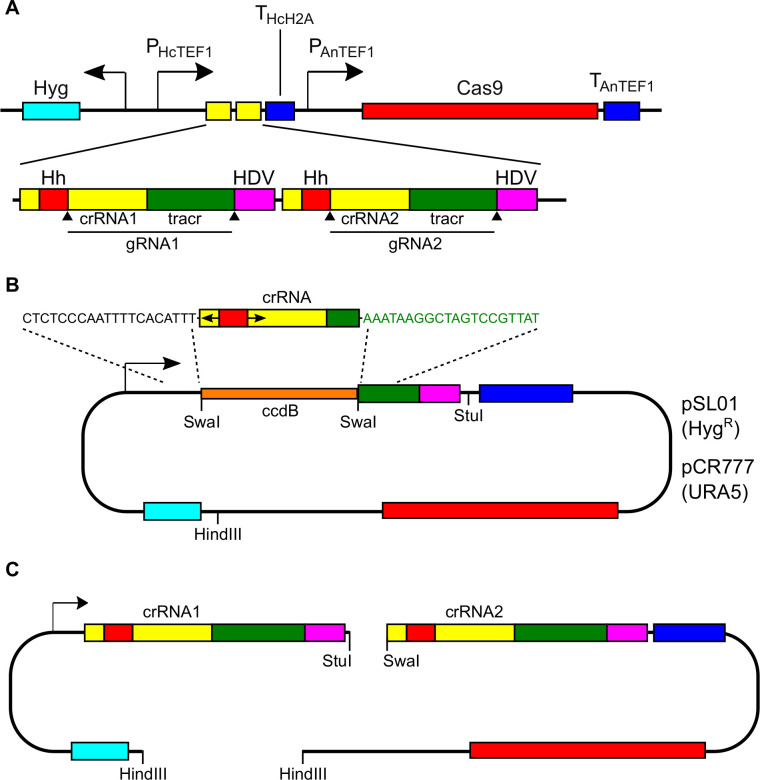
Dual gRNA CRISPR/Cas vectors designed for the generation of targeted gene deletions. (A) Schematic representation of a CRISPR/Cas9 vector with an expression of tandem gRNAs controlled by the *Histoplasma TEF1* promoter and *H2A* terminator. Each gRNA cassette includes the crRNA and the tracrRNA flanked by sequences of the hammerhead (Hh; red) and the HDV ribozyme sequences (purple) for processing of the RNA into the appropriately sized gRNA. (B and C) Schematic representation of the construction of single gRNA-containing vectors and the combination of two single gRNA vectors into the dual gRNA vector. (B) Single-target CRISPR/Cas9 plasmids are constructed in vectors pSL01 (hygromycin resistance selection) or pCR777 (uracil prototrophy selection) using homology-based ligation-independent cloning. The donor fragment consists of the 20-nucleotide crRNA sequence (yellow) and the 5′ portion of the tracrRNA (green). Upstream of the crRNA is the Hh ribozyme sequence (red) preceded by six nucleotides complementary to the 5′ 6 nucleotides of the crRNA (arrows) to enable base pairing necessary for the Hh ribozyme-mediated RNA cleavage. The crRNA:tracrRNA sequences are flanked by at least 20 nucleotides of the *Histoplasma TEF1* promoter (black nucleotides) and the 3′ portion of the tracrRNA (green nucleotides) homologous to the cut vector to enable recombination. (C) Synthesis of the dual gRNA vector is accomplished by digestion of single-target gRNAs with *StuI + HindIII* or *SwaI + HindIII* restriction enzymes and ligation of cohesive ends.

For loss of function studies, the generation of large deletions in the coding sequence (CDS) is preferable to small indel mutations, both because the resulting truncated gene is more likely to have lost function and because large genomic deletions are less likely to revert. In addition, large deletions can easily be detected by PCR of the target region, whereas small indels can only be detected by DNA sequencing, which is slower and more expensive. Kujoth et al. (26) showed that CRISPR/Cas9-based gene editing events could be generated simultaneously in two genes in *Blastomyces* by the expression of two gRNAs. Expression of two separate gRNAs targeting the same gene could potentially induce deletion of the sequence between the targeted sites. To this end, we constructed a dual gRNA vector with tandem gRNA cassettes driven by the *Histoplasma TEF1* promoter (Fig. 4A). Each crRNA cassette has its own RNA processing signals [hammerhead and hepatitis delta virus (HDV) ribozymes] to produce the appropriately sized gRNAs (Supplemental Figure 2). To construct this vector, 20 nucleotide protospacer sequences are individually cloned into a *SwaI*-digested gRNA vector (pSL01 or pCR777) by ligation-independent cloning mediated by the inclusion of sequences upstream and downstream of the *SwaI* sites (Fig. 4B). The two single-target gRNA vectors are then combined by restriction-enzyme and ligation-mediated cloning, placing the two gRNA cassettes in tandem by ligation of the *StuI* and *SwaI* blunt-cut DNA (Fig. 4C).

**Fig 5:**
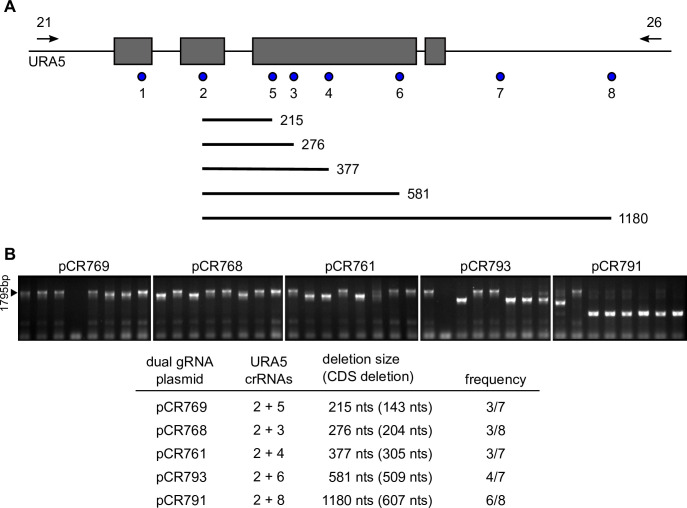
Dual *URA5*-targeting sgRNA CRISPR/Cas9 vectors generate deletions of the *URA5* gene. (A) Schematic representation of the *Histoplasma URA5* locus and locations of the *URA5*-targeting protospacer sequences of the crRNAs. The *URA5* genome context is shown with exons (gray rectangles) and the locations of the crRNAs (blue circles). Potential deletions from combinations of the *URA5* crRNAs are shown below the gene with numbers indicating the distance separating the two crRNAs. Primers (*URA5*-21 and *URA5*-26; arrows) for screening of genomic DNA for deletions are shown above the locus. (B) Recovery of *URA5* deletion mutants. Individual clonal Ura^−^ lines (*n* = 8 for each) were tested by PCR for the size of the *URA5* locus with smaller PCR products (<1,795 base pairs; arrowhead) indicating deletions in the *URA5* gene. Sizes of PCR products were determined by gel electrophoresis. The table shows frequencies of deletions of the *URA5* gene induced by the CRISPR/Cas9 dual gRNA vectors in *Histoplasma* yeasts with the number of deleted bases of the CDS listed in parentheses.

Although two gRNAs could be simultaneously expressed, it was unclear if their expression would result in deletion of the intervening sequences or merely creation of separate indel mutations. To test this, we used two crRNAs targeting the *URA5* gene so that mutational events could be selected using 5-fluoroorotic acid (5-FOA), and the resultant Ura^−^ mutants screened for deletions. Eight different *URA5*-targeting crRNAs were designed across the *URA5* gene and downstream region to test whether there was a separation limit to the generation of deletions by repair of separate CRISPR/Cas9-based double-strand DNA breaks (Fig. 5A). Dual gRNA vectors were constructed using *URA5* crRNA#2 in combination with other *URA5* crRNAs separated by increasing length. Transformation of these gRNA constructs into *Histoplasma* yeasts followed by plating of the transformed yeast on media with 5-FOA produced many 5-FOA-resistant (Ura^−^) transformant colonies. PCR performed on eight independent transformants from each dual gRNA construct-mediated uracil auxotroph using primers that flank the *URA5* locus showed most transformants produced smaller PCR products whose size was consistent with deletion of the sequences between the specific crRNA target regions of the *URA5* locus (Fig. 5B). Sequencing of a subset of the shorter PCR products confirmed deletion mutations were bordered by the crRNA target sites. There appears to be no correlation between deletion frequency and the size of the separation of the crRNA targets, at least up to 1,180 base pairs. Thus, simultaneous expression of two gRNAs enables recovery of deletion mutants, which can be quickly determined by PCR of transformants.

To validate the broader applicability of the system in *Histoplasma*, we used the CRISPR/Cas9 system to generate *URA5* mutants in diverse *Histoplasma* clinical isolates. These isolates belong to distinct clades/species originally designated by Kasuga et al. (30), which have been subsequently designated as separate species (27). In addition to the G217B North American type 2 (NAm2)/*Histoplasma ohiense* strain used in optimization experiments above, *Histoplasma* isolates were obtained, which represented the North American type 1 (NAm1)/*H. mississippiense* group (e.g., strains WU24 and HC17, and HC30), and Latin American (LAmA)/[*H. suramericanum* species (strain HC20); Supplemental Figure 1]. Both single and dual gRNA systems were transformed into these isolates, and Ura^−^ transformants were selected using 5-FOA selection. Mutants were confirmed as uracil auxotrophs by culture (data not shown), and sequencing of the *URA5* gene showed each mutant harbored a mutation in the *URA5* locus (Table 1). This confirms that the high frequency of gene editing with this system is not due to a unique feature of the G217B background. Furthermore, as *ura5* mutants, these *Histoplasma* strains are now amenable to molecular genetics using common *URA5*-based *Histoplasma* expression and RNAi vectors to expand studies to wide ranging *Histoplasma* isolates.

**TABLE 1:** CRISRP/Cas-generated *Histoplasma* mutant strains

Strain	Background^*a*^	crRNA protospacer sequence	Locus: mutation^*b*^
OSU415	G217B (NAm2/*H. ohiense*)	GTCTCTCCGCCAATGTGCTG	*URA5*: +1 @ nt 68
OSU416	G217B (NAm2/*H. ohiense*)	TTCCGCTATTTCTACCGCCT	*URA5*: +3 @ nt 171
OSU417	WU24 (NAm1/*H. mississippiense*)	GTCTCTCCGCCAATGTGCTG	*URA5*: +1 @ nt 68
OSU418	Hc17 (NAm1/*H. mississippiense*)	GTCTCTCCGCCAATGTGCTG	*URA5*: +1 @ nt 68
OSU419	Hc20 (LAmA/*H. suramericanum*)	TTCCGCTATTTCTACCGCCT	*URA5*: −2 @ nt 172
OSU420	Hc30 (NAm1/*H. mississippiense*)	TTCCGCTATTTCTACCGCCT	*URA5*: −1 @ nt 172
OSU427	Hc30 (NAm1/*H. mississippiense*)	TTCCGCTATTTCTACCGCCT, GAAGCCAAGGATCACGGAGA	*URA5*: Δ 171–374 (Δ 245–519 CDS)
OSU439	G217B (NAm2/*H. ohiense*)	GAAGCAGTCCATGTCAAGCG GGTCGGAGATTCGAGGCCAG	*CTR3*: Δ 26–534 (Δ26–367 CDS)
OSU442	G217B (NAm2/*H. ohiense*)	TTCCGCTATTTCTACCGCCT, GCGAGAGACGTTAAAGTTAG	*URA5*: Δ 171–475 (Δ 244–620 CDS)

^*a*^ Clinical isolate with phylogenetic group/species, as defined in references (30) and (27), in parentheses.

^*b*^ Type of indel or deletion at the position relative to the URA5 or CTR3 CDS start.

### Optimized workflow for generating deletions in target genes

**Fig 6:**
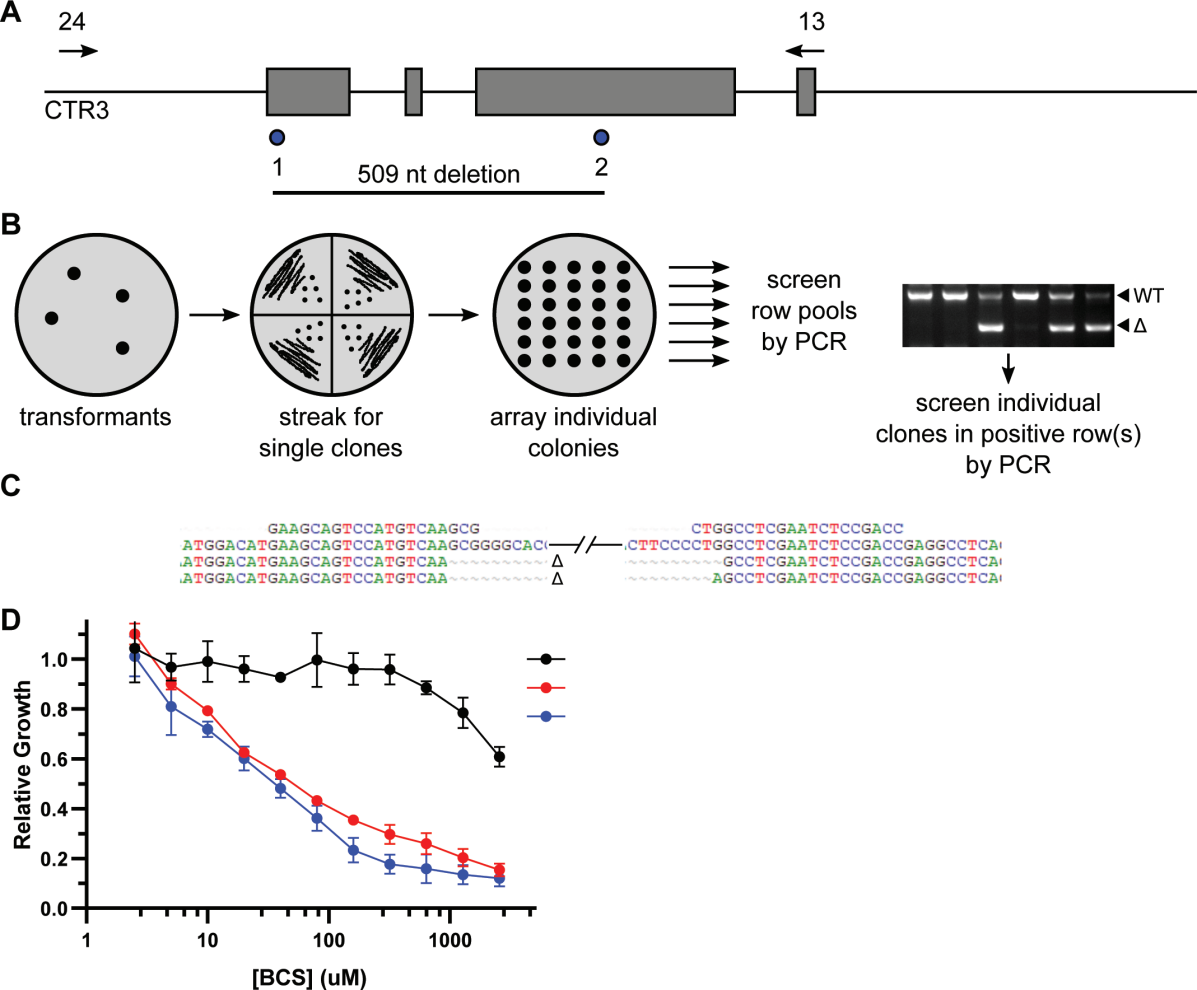
CRISPR/Cas9 generates deletions of the *CTR3* gene. (A) Schematic representation of the *Histoplasma CTR3* locus and locations of the *CTR3*-targeting crRNAs. The *CTR3* genome context is shown with exons (gray rectangles) and the locations of the protospacer sequences of the crRNAs (blue circles) and the putative deletion of the intervening sequence. Primers (*CTR3*-12 and *CTR3*-13; arrows) for screening of genomic DNA for deletions are shown above the locus. (B) Procedure for the recovery of deletion mutants from pool screening. CRISPR/Cas9 vector transformants are streaked to isolate clonal populations from which single colonies are arrayed on a solid medium master plate. Portions of each patch in a row are pooled to represent each row, DNA is isolated from each pool, and the pool is screened by PCR for products smaller than the wild-type (WT) PCR amplicon. Individual clones from a positive row are screened secondarily by PCR to identify the individual clone with a deletion mutation. (C) Generation of deletion mutants at the *CTR3* gene. A dual gRNA vector targeting the *CTR3* gene was used to isolate candidate deletion mutants and two recovered mutants sequenced to confirm the mutation. Two isolates (C1 and F3) showed deletions bordered by the cut sites defined by the two *CTR3* crRNAs. (D) Confirmation of the loss of Ctr3 function. The two deletion mutants were tested for the ability to grow in increasing concentrations of the copper-chelator BCS. Curves represent growth of the *ctr3* deletion mutants (red and blue) relative to wild-type yeasts (*CTR3*, black). Data points represent the relative growth of biological replicates (*n* = 3).

To determine the applicability of this dual gRNA system to generate deletions in target genes without selection or visible mutant phenotypes, we tested its ability to produce deletions in the *CTR3* gene, which is required for the growth of *Histoplasma* in copper-restricted environments (31, 32). Two protospacer elements representing sequences located in the first and third exons of the *CTR3* gene were designed, which would produce a 509base pair deletion eliminating 55% of the *CTR3* CDS (Fig. 6A). Since mutants in the *CTR3* locus cannot be directly selected, we adopted a pool screening approach to reduce the number of DNA isolations necessary to prepare for PCR (Fig. 6B). Individual transformants were picked and streaked on solid media to generate clonal populations from the transformation selection plate. Single colonies (at least eight) from a given transformant line were arrayed on fresh solid media. After growth, cells from each patch in a row were combined and DNA isolated from the pool. The row pools were screened by PCR using *CTR3* primers flanking the intended deletion. Both full-length and deletion-sized PCR products were found among the transformants and used to identify rows with clones harboring the deletion mutation. The clones in such positive rows were subsequently screened individually by PCR to identify the deletion mutant. Since the CRISPR/Cas9 system was built on a *URA5*-based telomeric plasmid design, once identified, the desired *ctr3* deletion mutant was “cured” of the CRISPR/Cas9 plasmid by passaging in uracil-containing media. Two mutants (C1 and F3) were recovered, and sequencing showed independent CRISPR/Cas9-mediated deletion events that differed by one base pair at the right boundary (Fig. 6C). As predicted, growth of the two mutants in media containing increasing concentrations of the copper-chelator bathocuproine disulfonate (BCS) confirmed that the *ctr3* deletion mutants showed reduced growth in copper-restricted media (Fig. 6D) similar to that of other loss-of-function mutants of *CTR3* (31). Although the CRISPR/Cas9 plasmid is episomal, to check for unanticipated chromosomal integration in “cured” strains, the loss of uracil prototrophy was confirmed by culture on a medium lacking uracil (data not shown), and PCR was performed on the *ctr3* and one of the *ura5* deletion mutants (Fig. 5) to confirm loss of regions of the respective CRISPR plasmids (Supplemental Figure 3).

## DISCUSSION

In this study, we have established optimized vectors and methodology for producing deletion mutants in targeted genes using a vector for expression of dual gRNAs. Generation of deletion mutants is a preferred approach for loss-of-function studies provided that the gene of interest can be targeted. While small indels can cause loss of function, one-third of indels (those inserting/deleting multiples of three nucleotides) that keeps the coding sequence in-frame may not significantly impact protein structure, and it is difficult to predict. Frameshift mutations are more likely to eliminate gene product function, but revertant indels can be produced in cells under selection, restoring some or all of the gene function. Large deletions of the coding region are likely to produce true loss-of-function phenotypes and lack the possibility of revertant mutations arising in the coding region. In addition, deletions facilitate more efficient screening of CRISPR/Cas9-mediated events by PCR rather than DNA sequencing.

The optimized CRISPR/Cas9 system for *Histoplasma* produces a high frequency of mutation events in a targeted locus. For single gRNAs, the frequency of mutation can be greater than 90% (Fig. 3C). We showed that the choice of the gene transcription regulatory elements was important for enhancing the frequency with stronger transcription initiation and efficient termination being critical. We used Pol(II) instead of Pol(III) promoters as Pol(III) promoters have not been defined in *Histoplasma*. The ability to use a strong Pol(II) promoter (P*_TEF1_*) to drive the expression of the gRNA is one advantage of this system since Pol(III) promoters may not produce as much gRNA as Pol(II) and would be less effective. As found in other systems, the protospacer sequence of the crRNA also impacts the efficiency of gene editing, but optimized search tools help simplify the identification of good crRNA sequences. The frequency of recovered mutants for the *ura5* and *ctr3* deletion strains was lower than recovery of mutations generated through single-target gRNA expression. Although the genes targeted for mutation were different (GFP versus *URA5* or *CTR3*), a more likely explanation is that the decrease in efficiency in the dual gRNA system is due to the requirement for two DNA break events to occur within the same chromosome in the same cell. We suspect that the sequence context of each DNA determined by the protospacer and surrounding nucleotides needs to be similar in order to obtain deletion mutants over mutants with two separate indels. Nevertheless, the frequency of deletion mutants was sufficient to enable recovery of mutants from the screening process with reasonable effort. Furthermore, the system described and the efficiency of mutant generation does not require a selection marker to be incorporated with the mutation, resulting in rapid isolation of marker-less deletion alleles. Once constructed, the CRISPR/Cas system is easily removed from mutant strains by virtue of its encoding on an episomal telomeric vector. Nonetheless, the CRISPR/Cas vector could potentially integrate into the chromosome causing unsuspected mutations or phenotypic changes to the strain. Thus, loss of the CRISPR/Cas vector in isolated mutants cured of the CRISPR/Cas vector should be validated. Ideally, whole genome sequencing can be performed, or more practically, loss of the vector selection marker by microbiological culture or loss of vector sequences by PCR (Supplemental Figure 3) can be done. The ability to generate marker-less deletions and curing strains of the specific gene-targeting vector facilitates the construction of deletions in multiple genes through successive application of the CRISPR/Cas system.

CRISPR/Cas9 provides an efficient method for generating targeted gene deletions for functional studies of genes. Previous means of producing genetic mutants in *Histoplasma* in targeted genes were plagued by inefficiencies or long procedures for recovery of the desired mutant. Although successful in some *Histoplasma* strains, directed deletions by reliance on allele replacement are inefficient due to the very low rates of homologous recombination. Passaging transformants repeatedly can increase the recovery of homologous recombination events but adds substantial time and has variable rates of success. Insertional mutagenesis mediated by *Agrobacterium* transformation is more efficient in generating loss-of-function mutants, and pools can be screened for insertions in a desired target gene if a selectable phenotype is available (21). However, subsequent recovery of the mutant from large mutant pools can be laborious and time-consuming given the slow growth rate of *Histoplasma* yeasts. RNAi is a faster way to deplete gene functions in *Histoplasma* (17, 31 - 40), which typically results in strains that phenocopy loss-of-function mutants (16, 17, 31, 35, 40). However, RNAi may be unstable under selection. Thus, a genetically stable, non-revertable gene deletion is preferable, and this can now be reliably produced in *Histoplasma* using CRISPR/Cas9. The procedure we describe with the optimized dual-sgRNA-Cas9 vector results in recovery of a deletion mutant in 30–40 days:

- 8–10 days for the initial transformation of yeasts with the dual gRNA CRISPR/Cas9 plasmid
- 8–10 days for picking single colonies from the transformants
- 4–5 days for growth of arrayed clones
- 1 day for DNA isolation and PCR screening of row pools
- 1 day for screening individual clones
- 8–12 days for passaging of mutants to cure them of the CRISPR/Cas9 plasmid

While efficient, this CRISPR/Cas9 system for *Histoplasma* has so far only been exploited for the generation of either indels (26, 28, 35) and now gene deletions. The location of the mutations depends on the locations of efficient CRISPR protospacer sequences in the genes of interest. Some CRISPR/Cas9 systems have been developed for allelic replacement with the Cas9-generated DNA break stimulating recombination with a separately provided replacement allele (4, 8). This would enable introduction of transgenes or designed mutations at a native locus. However, we suspect that the predominance of non-homologous end joining in *Histoplasma* at the expense of homologous recombination may need to be overcome for this next step in utilization of CRISPR/Cas9 gene editing. For now, CRISPR/Cas9-mediated gene deletions represent the best approach to enable functional studies of genes to probe the biology and pathogenesis of *Histoplasma*.

## MATERIALS AND METHODS

### Construction of gRNAs with gene-targeting crRNAs

CRISPR gRNAs were designed against GFP and the *Histoplasma URA5* and *CTR3* genes using CRISPOR [(41), http://crispor.tefor.net/crispor.py] using the gene sequence as input and selecting the *Histoplasma* strain G217B as the genome sequence and the NGG SpCas9 as the Protospacer Adjacent Motif. Candidate protospacer element sequences were selected with high Chari scores (>60), high Doench “16 scores”(>65), and high Wang scores (>70). Both forward and reverse direction protospacer sequences were used. DNA sequences with the protospacer element were incorporated into crRNA and were synthesized into short gene fragments (Twist Biosciences). Gene synthesis included the hammerhead ribozyme sequence preceded by six bases complementary to the 5′ end of the crRNA necessary for RNA duplex formation and ribozyme-based cleavage, the 20-nucleotide crRNA sequence, and the 5′ half of the tracrRNA. The synthesized DNA also included at least 20 nucleotides matching the promoter and the 3′ end of the tracrRNA to facilitate ligation-independent cloning.

The synthesized DNAs containing the crRNA sequences were cloned into the *SwaI*-digested CRISPR/Cas9 telomeric vector using In-Fusion cloning (Takara) and transformation into DH5ɑ *Escherichia coli*. For dual gRNA vectors, single-target gRNA vectors were combined by digesting one vector with *HindIII* and *StuI* and the second with *HindIII* and *SwaI*. Fragments were gel purified and ligated with T4 DNA ligase and transformed into DH5ɑ *E. coli*.

### *Histoplasma* transformation and culture

The *Histoplasma* strains used in the optimization of the CRISPR/Cas9 vector were derived from the North American clade 2*/H*. *ohiense* clinical isolate G217B (ATCC 26032). Clinical strains and their derived uracil-auxotrophs representing other phylogenetic clades/species are listed in Table 1 and Supplemental Figure 1. All experiments were done with *Histoplasma* yeasts grown in *Histoplasma*-macrophage medium (HMM) (42) with continuous shaking (200  rpm) at 37°C. For growth on solid media, HMM was solidified with 0.6% agarose and the media supplemented with 25 µM FeSO_4_. Uracil (100 µg/mL) was added to HMM for the growth of uracil auxotrophs. Yeasts in exponential growth were transformed with CRISPR/Cas9 vectors or with promoter-GFP fusion vectors by electroporation (43), following linearization of the telomeric vectors with *PacI*. Ura^+^ transformants with *URA5*-based vectors were selected on HMM. For the generation of uracil auxotrophs, yeasts were transformed with a CRISPR/Cas9 vector with the hygromycin-phosphatase gene, and transformants were selected on HMM supplemented with uracil, hygromycin B (80 µg/mL), and 5-FOA (1 mg/mL). For passaging of yeast cells, yeasts were diluted 1:50 into fresh HMM when cultures reached saturation as measured by optical density at 600 nm. For curing yeasts of the CRISPR/Cas9 vector, yeasts were sub-cultured in liquid media without selection (i.e., HMM with uracil).

### Transcriptional regulatory sequences

*Aspergillus* promoter sequences (*GPD* and *TEF1*) and upstream regions of the *Histoplasma TEF1* and *H2B* genes (657 bp and 638 bp, respectively) were amplified by PCR and cloned into the *URA5*-based *Histoplasma* GFP-reporter T-DNA vector pCR711. The putative terminator region of the *H2A* gene consisting of the 684 base pairs immediately downstream of the *H2A* coding sequence was amplified by PCR for cloning into the CRISPR/Cas9 vector. GFP reporter plasmids were transformed into *Histoplasma* using *Agrobacterium*-mediated transformation (44).

### PCR-based screening for CRISPR/Cas9-mediated deletions

Single colonies were picked and arrayed on HMM. Portions of the resultant patches in each row were picked into a tube with 100 µL of H_2_O. To isolate DNA, 100 µL of 2× lysis solution (2% Triton X-100, 20 mM EDTA), approximately 100 µL of glass beads (0.5 µm diameter), and 100 µL of phenol:CHCl_3_:isoamyl alcohol (25:24:1) were added to the suspended cells, and the yeasts disrupted by beating the tubes for 2 min. DNA was recovered by centrifugation of the lysate (16,000 × *g* for 5 min) and transferring 50 µL of the aqueous (upper) phase into 200 µL of H_2_O. This crude DNA-containing solution was used as the template (2% vol/vol) for PCR using Taq polymerase and primers flanking the potential gene deletion. Clonal patches in rows with deletion-harboring clones were tested individually by PCR. Primers used for screening of deletions are given in Supplemental Table 1. Deletions were confirmed by Sanger sequencing of the PCR products generated from purified mutants.

### Phenotyping of CRISPR/Cas9-generated mutants

GFP fluorescence of yeast colonies was visualized using a modified gel documentation system (18) with fluorescence excitation (470 nm) and emission filters (530 nm). Fluorescence was quantified using Fiji/ImageJ (45). Uracil auxotrophy was verified by testing the dependence on uracil supplementation for growth in HMM. To confirm the loss of Ctr3 function, yeasts were tested for the ability to grow in limited copper using liquid HMM containing a twofold dilution series of the copper-chelator BCS. Growth was normalized to the growth of yeasts in the absence of BCS.

### Phylogenetic classification of *Histoplasma* strains

Clinical isolates were assigned to phylogeographic clades/species based on nucleotide polymorphisms in four protein-coding genes [alpha tubulin (*TUB1*), Δ9-fatty acid desaturase (*OLE1*), ADP-ribosylation factor (*ARF1*), and H-antigen/β-glucosidase (*HAG1*)]. The loci were amplified from genomic DNA from each strain and sequenced. Phylogenetic relationships were determined among the clinical isolates and sequences of *Histoplasma* strains representing each phylogenetic group (27, 30). The four gene sequences were concatenated and aligned using Clustal Omega (46). The maximum likelihood phylogenetic tree was generated using MEGA alignment software [version 11; reference (47)].

## SUPPLEMENTAL MATERIAL

Supplemental Figures and Table msphere.00178-23-s0001.pdf Figures S1 to S3 and Table S1.

ASM does not own the copyrights to Supplemental Material that may be linked to, or accessed through, an article. The authors have granted ASM a non-exclusive, world-wide license to publish the Supplemental Material files. Please contact the corresponding author directly for reuse.
