# Supplementary material for: Targeted gene deletions in the dimorphic fungal pathogen Histoplasma using an optimized episomal CRISPR/Cas9 system
Source: mSphere. 2023 Jun 30;8(4):e00178-23. doi: 10.1128/msphere.00178-23 (PMC10449496; doi:10.1128/msphere.00178-23)
Supplement: Supplemental Figures and Table — Figures S1 to S3 and Table S1. [file msphere.00178-23-s0001.pdf]

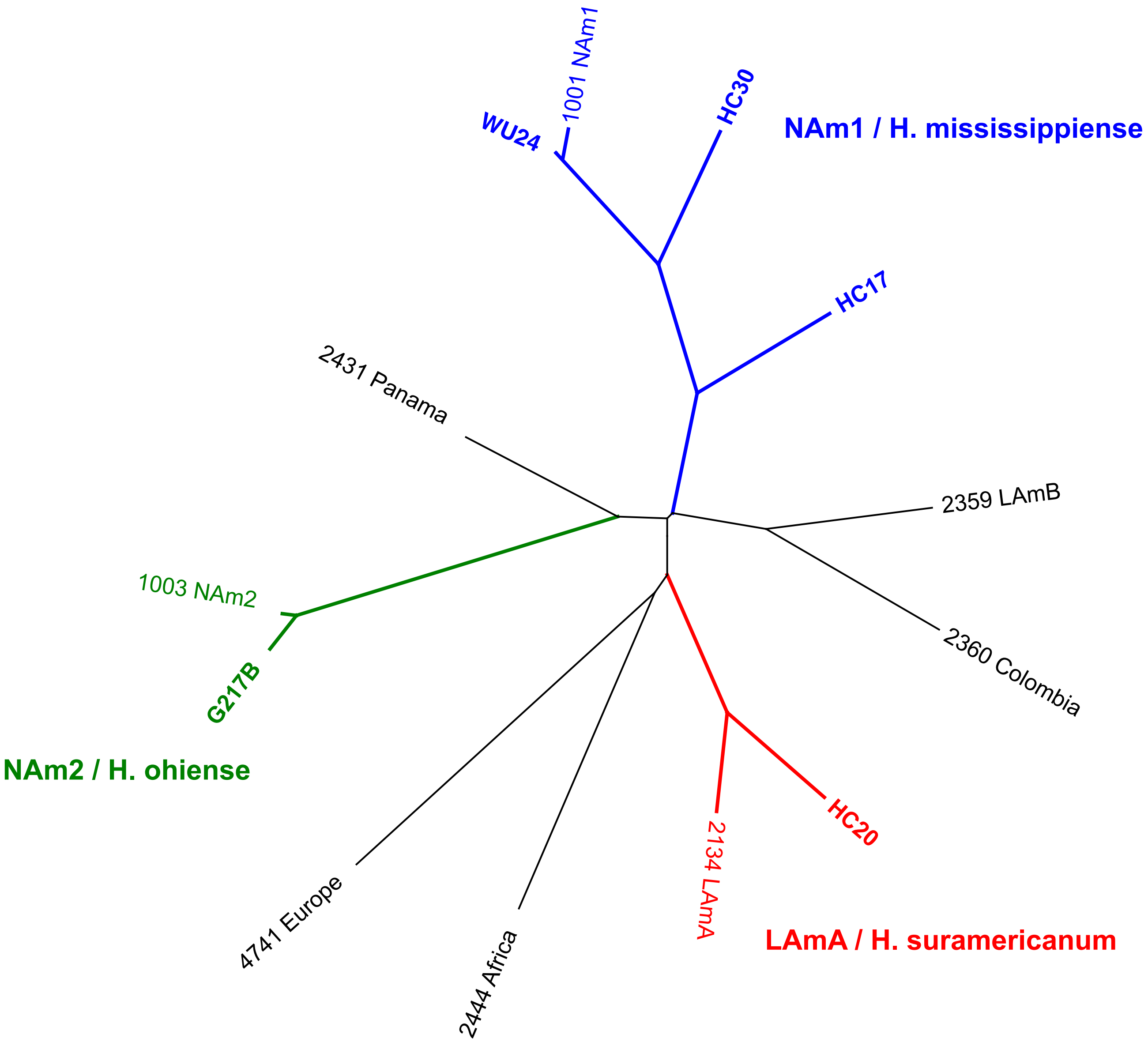

Supplemental Figure 1. Phylogenetic group/species assignment of *Histoplasma* strains. *Histoplasma* isolates G217B, HC17, HC20, and HC30 belong to the North American type II (NAm2)/*H. ohiense* (green), North American type I (NAm1)/*H. mississippiense* (blue) and Latin American type A (LamA)/*H. suramericanum* (red) clades/species based on relatedness to the type strains 1003, 1001, and 2134, respectively. The maximum-likelihood unrooted phylogenetic tree was assembled from four-locus sequencing (*TUB1, OLE1, ARF1*, and *HAG1* genes) using MEGA phlylogenetic software (v11) and named following Kasuga T, et al. (2003) and Sepulveda V, et al. (2017).

5'-ctctcccaattttcac<u>attt</u>**AAATCCCGCCACCNNNNNNCTGATGAGTCCGTG AGGACGAAACGAGTAAGCTCGTC*NNNNNNNNNNNNNNNNNNNNGTTTTAGAGCTA GAAATAGCAAGTTA**<u>aaat</u>aaggctagtccgttatcaacttgaaaaagtggcaccga gtcggtgctttt*ggccggcatggtcccagcctcctcgctggcgccggctgggcaa catgcttcggcatggcgaatgggac-3'

P_{TEF1} promoter fragment
Hammerhead ribozyme
crRNA
tracrRNA
HDV ribozyme

Supplemental Figure 2: Sequence of the gRNA cassette and synthesis of gene-specific inserts. The crRNA (yellow highlight) containing the protospacer sequence (N_{20}) and the tracrRNA sequence (green highlight) are flanked by the Hammerhead (red text) and HDV (purple text) ribozyme sequences with ribozyme cleavage sites denoted with asterisks. Six bases complementary to the 5' end of the protospacer sequence are added 5' of the Hammerhead ribozyme sequence to facilitate base paring and precise cleavage of the RNA at the beginning of the protospacer. The synthesized sequence (bold text) is cloned into the SwaI cut vector (SwaI half sites are indicated by underlined text) using ligation-independent cloning by inclusion of 20 nucleotides homologous to the upstream P_{TEF1} promoter and downstream 3' tracrRNA sequence of the vector.

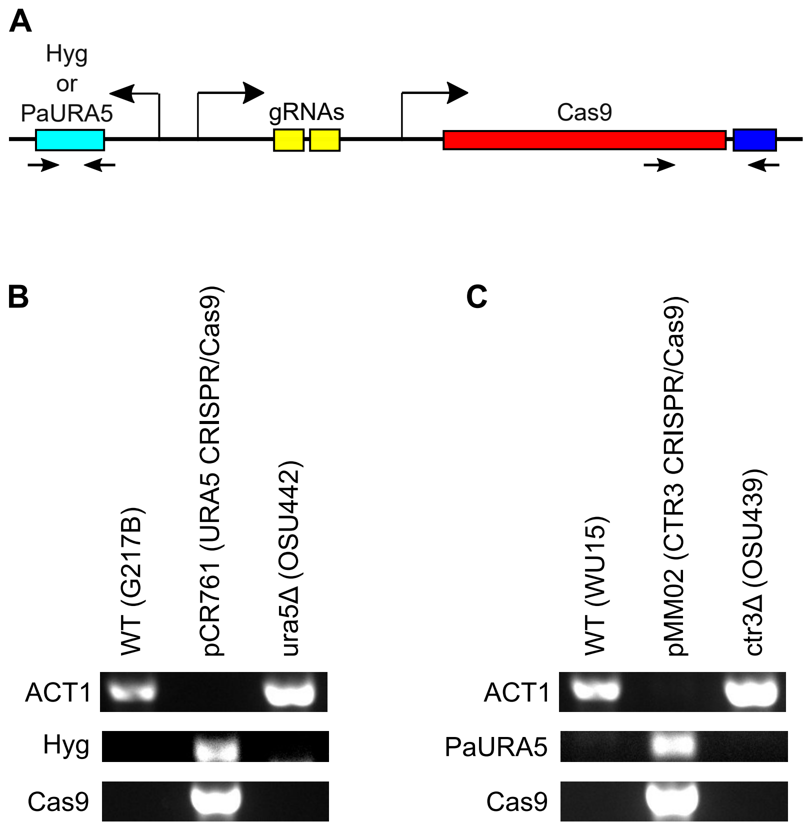

D

Region	primer 1	primer 2	annealing temp	amplicon size (bp)
ACT1	ACT1-5 (GGTTTCGCTGGCGATGATGCTC)	ACT1-6 (GGACTCGTCGTACTCCTGCTTCG)	54°C	1276 bp
Hyg	HPH-5 (CACGTTGCAAGACCTGCCTG)	HPH-9 (CTATTCCTTTGCCCTCGGACG)	55°C	732 bp
PaURA5	URA5-17 (AGAAAGACTTCCTCAAATCCGC)	URA5-18 (CTCCCTATACTCCTCAGTCCGC)	54°C	646 bp
Cas9	CRISPR-1 (GTCGGTCAAGGAACTGCTCG)	ACT1-6 (CTAGTGACCTCCACTAGCATTACACTTG)	58°C	1175 bp

Supplemental Figure 3. Confirmation of loss of CRISPR/Cas9 vector from ura5 and ctr3 deletion mutants. (**A**) Generalized schematic of the CRISPR/Cas9 vector. Arrows indicate the location of PCR primer pairs used to amplify regions of the vector corresponding to the selection marker (Hygromycin resistance (Hyg) or the *Podospora anserina URA5* gene) and to the Cas9 coding sequence and terminator. (**B-C**) PCR amplification of regions corresponding to the *Histoplasma* chromosomal actin gene (*ACT1*) or the CRISPR/Cas9 vector regions corresponding to the selection maker or Cas9. PCR confirms loss of vector pCR761 sequences from the *ura5* deletion mutant (OSU442; **B**) or loss of vector pMM02 from the *ctr3* deletion mutant (OSU439; **C**). Parental *Histoplasma* strains (WT) used as the background for each deletion mutant are indicated, respectively. (**D**) PCR primers and annealing temperatures for each amplicon (size in base pairs).

Supplemental Table 1: Primers used for URA5 and CTR3 deletion screening

Primer	Sequence	Direction (rel to gene)
URA5-21	GCTCTAATACTGGCGATCCACTCC	forward
URA5-26	ATTACTTCCTAGGCGGCGGTCTGC	reverse
CTR3-12	GATGCCTAGGTAGGGATGCCAAC	forward
CTR3-13	GCCACAACATACGGTCACCT	reverse
